# Automatic inhibitory function in the human somatosensory and motor cortices: An MEG-MRS study

**DOI:** 10.1038/s41598-017-04564-1

**Published:** 2017-06-26

**Authors:** Chia-Hsiung Cheng, Shang-Yueh Tsai, Chia-Yih Liu, David M. Niddam

**Affiliations:** 1 Department of Occupational Therapy and Graduate Institute of Behavioral Sciences, Chang Gung University, Taoyuan, Taiwan; 2 Laboratory of Brain Imaging and Neural Dynamics (BIND Lab), Chang Gung University, Taoyuan, Taiwan; 3 Healthy Aging Research Center, Chang Gung University, Taoyuan, Taiwan; 4 Department of Psychiatry, Chang Gung Memorial Hospital, Linkou, Taiwan; 5 Graduate Institute of Applied Physics, National Chengchi University, Taipei, Taiwan; 6 Mind, Brain and Learning Center, National Chengchi University, Taipei, Taiwan; 7 School of Medicine, Chang Gung University, Taoyuan, Taiwan; 8 Institute of Brain Science, National Yang-Ming University, Taipei, Taiwan; 9 Brain Research Center, National Yang-Ming University, Taipei, Taiwan

## Abstract

While the automatic inhibitory function of the human cerebral cortex has been extensively investigated by means of electrophysiological recordings, the corresponding modulating neurochemical mechanisms remain unclear. We aimed to examine whether the primary somatosensory (SI) and primary motor cortical (MI) inhibitory function is associated with endogenous GABA levels. Eighteen young participants received paired-pulse and single-pulse electrical stimulation to the median nerve during magnetoencephalographic recordings. The SI sensory gating (SG), considered as an automatic inhibitory ability, was measured as the amplitude ratio of Stimulus 2 over Stimulus 1, in the paired-pulse paradigm. In addition, stimulus-induced beta activity, considered to originate from MI and also to be related to inhibitory function, was estimated using the single-pulse paradigm. The GABA+ concentration of the sensorimotor cortex was acquired from each subject by using magnetic resonance spectroscopy (MRS). A lower SG ratio in SI was significantly associated with an increased beta power in MI. More importantly, the beta rebound power, but not SI SG ratio, was positively correlated with GABA+ concentration. Our findings show a tight functional relationship between SI and MI during processing of automatic inhibition. GABA+ levels appear to be more closely related to the automatic inhibitory function of MI than SI.

## Introduction

Sensory gating (SG), the brain’s ability to pre-attentively filter out repetitive sensory information, is conceptualized as an automatic inhibitory function to protect the higher-hierarchical cortical centers from flooding of unnecessary sensory inputs^1–4^. The paired-stimulus paradigm, in which two identical stimuli are separated by 500 ms, has been extensively applied to study this type of cognitive function. The SG ability is typically measured as an amplitude ratio of responses to the second stimulus (S2) over responses to the first stimulus (S1). Theoretically, a lower ratio represents a better gating, i.e. inhibitory function^5, 6^. Although SG research has mainly focused on the auditory cortex, increasing evidence suggests that SG can be reliably evoked in the primary somatosensory cortex (SI) by paired-pulse electrical stimulation to the median nerve^3, 7–9^.

Electrical stimulation to the median nerve not only elicits phase-locked evoked responses, but also non-phase-locked brain oscillations, e.g., in the beta frequency range (13 to 30 Hz). The power of beta oscillations is substantially enhanced around 400 ms to 900 ms after the stimulus onset, but is dramatically reduced during concomitant hand movement^10–12^. Previous studies have shown that the neural generators of beta event-related desynchronization (ERD) following tactile stimulation were located in the somatosensory or sensorimotor cortex, while those of beta event-related synchronization (ERS, or rebound) following tactile stimulation or movement were located in the primary motor cortex (MI)^13–16^. Although not well understood, the motor cortical beta rebound is considered to be associated with a process of active immobilization or an increased transient motor cortical inhibition^17, 18^. Since the subjects do not have to respond to the stimulation, the beta rebound could be thought of as an automatic inhibitory function in MI.

Although cortical GABA levels play an essential role in the modulation of inhibitory processing, the underlying neurochemical mechanisms associated with SG and electricity-induced beta rebound oscillations are not well elucidated. The non-invasively recorded electroencephalographic (EEG)/magnetoencephalographic (MEG) responses are primarily generated by post-synaptic potentials, which are dependent upon the balance between excitatory glutamatergic principal cells and inhibitory GABAergic interneurons. In humans, the endogenous GABA concentration can be measured *in vivo* by means of magnetic resonance spectroscopy (MRS), a non-invasive neuroimaging technique. Since the GABA signal is often slightly contaminated by contributions from other molecules^19^, the combined signal is referred to as GABA+. A previous study suggests a positive relationship exists between the post-movement beta rebound (PMBR) and GABA+ levels in the sensorimotor (SM) cortex^16^. Moreover, the beta peak frequency at rest has also been associated with GABA+ levels in the SM cortex^20^. It remains, however, unknown whether a relationship exists between electricity-induced beta oscillations and GABA+ levels in the SM cortex and how these relate to the somatosensory SG ability.

The aim of the present study was to examine whether the automatic inhibitory function, measured by whole-head MEG, is modulated by the endogenous GABA levels. More specifically, we used MEG combined with electrical stimulation of the median nerve to obtain SI SG ratios and MI beta oscillatory activity. Furthermore, each subject’s resting GABA+ concentration in the SM cortex was acquired by MRS. We hypothesized that SI SG ratios were correlated with the MI beta rebound power due to intense reciprocal connections between these two regions. Based on previous studies, we also predicted that GABA+ levels would be positively correlated with the beta peak frequency and power. Finally, we expected a negative association between the SI SG ratios and SM cortical GABA+ concentration.

## Results

### Somatosensory SG ratio

**Figure 1:**
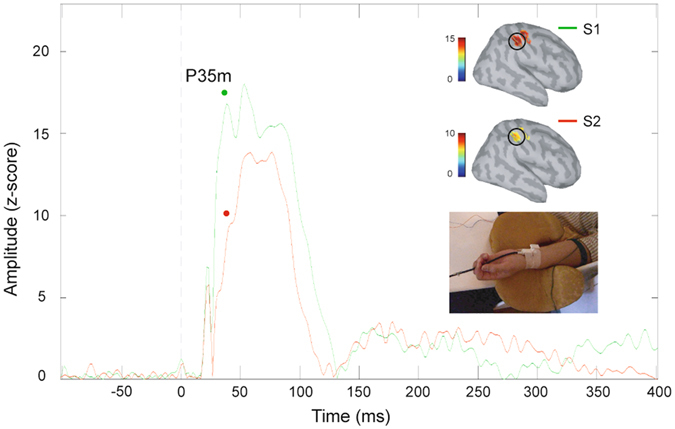
The grand-averaged source waveforms of P35m and the corresponding MNE maps in response to paired-pulse electrical stimulation. Responses to the second stimulus (S2, red trace) were dramatically suppressed compared to those to the first stimulus (S1, green trace), indicating a robust effect of sensory gating. SI, primary somatosensory cortex; MNE, minimum norm estimate.

The somatosensory SG ratio could be determined in all subjects (0.55 ± 0.04). Figure 1 shows the grand-averaged cortical maps of the P35m component and the SI source waveforms as a function of time. Cortical responses to S2 were significantly reduced compared to those of S2 in terms of the P35m component.

### MI beta oscillations

**Figure 2:**
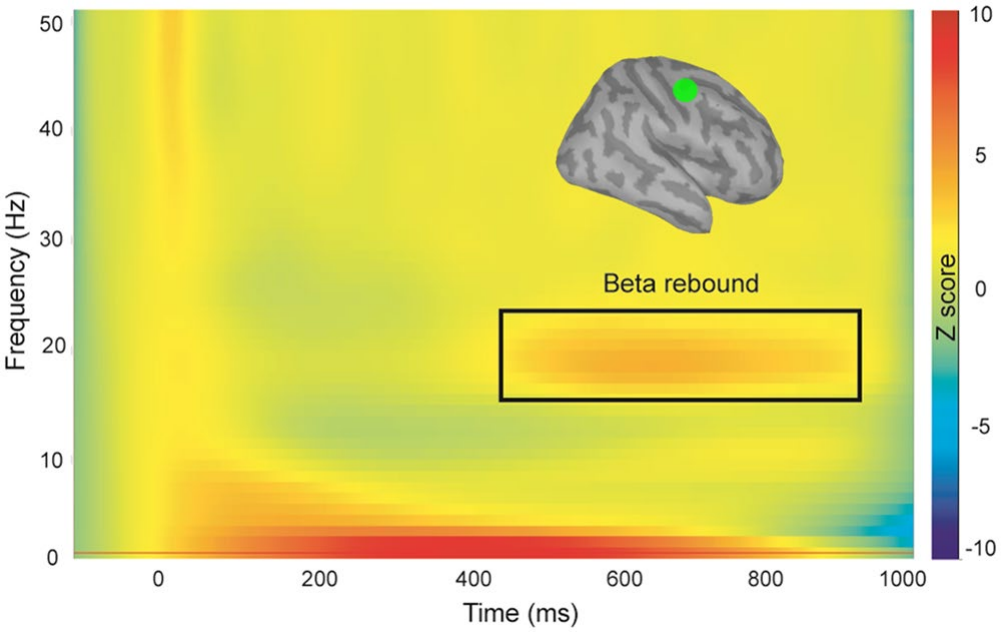
The grand-averaged time-frequency map of the contralateral right primary motor cortex (MI) for the frequency band of 1–50 Hz. A clear beta rebound (~20 Hz) was observed around 400–900 ms after the stimulus onset.

No obvious beta activity was detected in three subjects﻿﻿ (16, 17, and 18), resulting in 15 data points in the final analysis (Table 1). Figure 2 displays the grand-averaged time-frequency map over the time interval of −100 to 1000 ms and the frequency band from 1 to 50 Hz in the right MI. The strength of beta oscillations typically decreases immediately after median nerve stimulation, and then rebounds above the pre-stimulus baseline level at the time window of 400 to 900 ms. The mean strength of the beta rebound was 4.83 (SEM 0.76), with the most reactive frequency at 18.37 Hz (SEM 0.52).

**Table 1:** SG ratio, GABA+ level, and beta peak frequency and power in each subject.

Subject	SG ratio	Beta frequency	Beta power	GABA+ level
1	0.83	14.5	3.78	9.09
2	0.82	17.5	0.50	8.10
3	0.49	18.5	10.63	13.50
4	0.65	18.5	4.10	10.62
5	0.36	20.5	6.71	8.72
6	0.69	17.5	4.56	11.90
7	0.60	18.5	5.53	10.20
8	0.62	22.5	1.62	—
9	0.31	15.5	8.67	—
10	0.55	20.5	3.0	9.47
11	0.44	18.5	8.81	9.75
12	0.65	19.5	3.60	8.94
13	0.55	18.5	6.39	8.79
14	0.63	18.5	2.0	—
15	0.66	16.5	2.5	8.05
16	0.31	—	—	9.81
17	0.39	—	—	10.40
18	0.28	—	—	13.41
Mean	0.55	18.37	4.83	10.05
SEM	0.04	0.52	0.76	0.44

SG = sensory gating, SEM = standard error of the mean.

### GABA+ concentration

**Figure 3:**
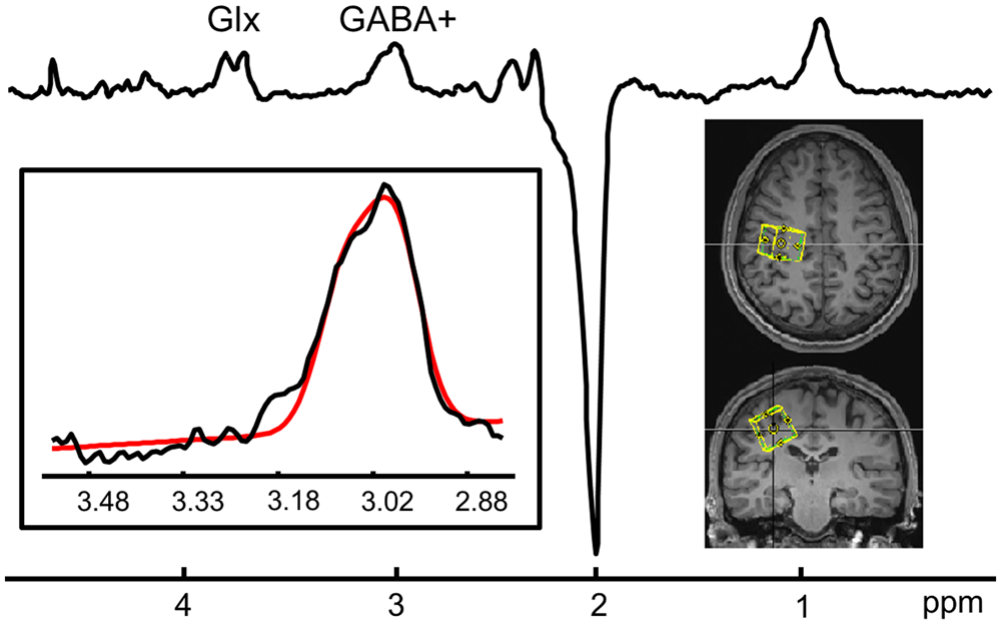
The voxel location covering the contralateral sensorimotor region and the edited spectra from a representative subject. The insert shows the fitted peak of GABA+ (red line) using two Gaussian models with 7 variables (2 height, width, splitting, baseline offset, and baseline gradient) in the spectral range of 2.79–3.55 ppm.

Figure 3 shows an example of VOI placement and the corresponding GABA+ spectra. Three subjects (8, 9, and 14) were excluded from the correlational analysis due to excessive head motion or failure to identify the GABA+ peak. The mean SM cortical GABA+ concentration was 10.05 (SEM 0.44) (Table 1).

### Correlation of MEG and MRS data

**Figure 4:**
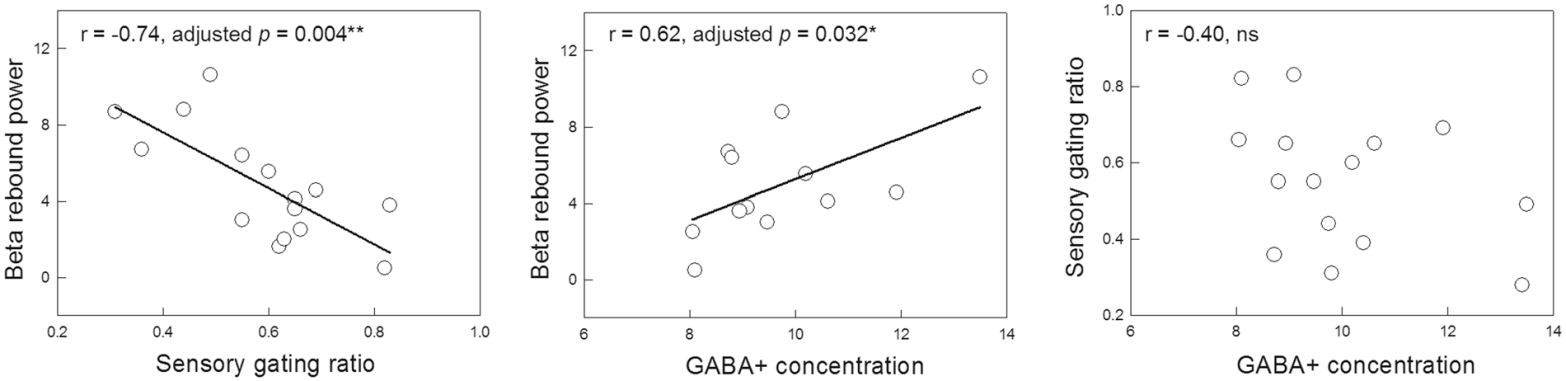
The SI SG ratio was significantly correlated with the MI beta rebound power. The GABA+ levels in the sensorimotor cortex modulated the variability of the MI beta rebound power, but not the beta peak frequency. No association was found between GABA+ concentration and the SI SG ratio.

Figure 4 demonstrates the correlations of MEG parameters and MRS-measured GABA+ level. As anticipated, the SI SG ratio was negatively correlated with the MI beta rebound power (Pearson’s r = −0.74, *p* = 0.004, one-tailed and adjusted; Spearman’s rho = −0.68, *p* = 0.012, one-tailed and adjusted), indicating that a better SG ability (lower ratio) is related to an increased motor cortical inhibition. Furthermore, the SI SG ratio, calculated from the absolute values of S1 and S2, was still significantly correlated with the MI beta rebound power (see supplementary information).

A positive association was observed between SM cortical GABA+ concentration and MI beta rebound power (Pearson’s r = 0.62, *p* = 0.032, one-tailed and adjusted; Spearman’s rho = 0.52, *p* = 0.08, one-tailed and adjusted). To further, examine the impact of the “high” GABA+ value of Subject 3, the analysis was conducted without this subject resulting in a non-significant finding (N = 11, r = 0.30, *p* = 0.187, one-tailed and adjusted). However, using the univariate Grubb’s test (see online calculator at: https://graphpad.com/quickcalcs/Grubbs1.cfm), no outliers were identified in our data.

Neither the SI SG ratio nor the MI beta peak frequency was correlated with GABA concentration (Pearson’s r = −0.40 and r = 0.07, respectively r; Spearman’s rho = 0.108 and rho = 0.091, respectively).

## Discussion

The primary goal of the present study was to investigate whether the automatic inhibitory function, as indexed by the SI SG ratio and the MI beta rebound activity, would be modulated by the endogenous GABA+ concentration in the same part of the cerebral cortex. Our results showed that the SI SG ratio was significantly associated with the MI beta rebound power. In addition, the GABA+ concentration was positively correlated with the beta rebound power, but not with the beta peak frequency. No association was observed between the SI SG ratio and GABA+ concentration.

The major finding of this study was the linear relationship between the MI beta rebound power and the GABA+ concentration. Previous studies using pharmacological modulation have shown that increases of resting neural oscillations in the beta frequency range result from increased GABA activity^21, 22^. However, other studies have shown that an elevated endogenous GABA level was associated with reduced movement-related beta power, i.e., PMBR^23^. Gaetz and colleagues, by using MEG and MRS, found that increases in motor cortical PMBR power was related to an increased GABA+ concentration^16^. The inconsistent results regarding the relationships between GABA level and beta power might be due to the differential effects of various subclasses of selective drugs (e.g., tiagabine, benzodiazepine, etc.) on the GABA concentration. In addition, it cannot be ruled out that the different tasks used to induce beta oscillations have an impact, i.e. where most of the previous studies examined movement-induced beta activity; we studied stimulus-induced beta activity. From a clinical perspective, it is noteworthy that, in contrast to movement-induced beta oscillations, electricity-induced beta activity bears less inter-individual variability and do not require overt behavioral responses. This provides a better clinical potential in particular when studying patients with movement disorders.

The relationship between the MRS-measured GABA+ concentration and MEG-recorded brain oscillations (i.e., gamma or beta) remains a matter of debate. Two pioneering studies revealed a robust relationship between GABA+ level and gamma oscillatory frequency in the primary visual cortex^24, 25^, whereas a recent study with a substantially larger number of participants did not find such a relationship^26^. In this context, several issues are worth considering. Firstly, Cousijn *et al*.^26^ applied short-echo-time MRS, while both Muthukumaraswamy *et al*.^25^ and we used the MEGA-PRESS technique. Gaetz *et al*.^16^, who investigated the motor cortical gamma and beta oscillations, also applied MEGA-PRESS and found an association between PMBR and GABA+ level in the sensorimotor cortex. Although we examined the electricity-induced beta rebound, our study parallels that of Gaetz *et al*. Secondly, we only observed an association of GABA+ level with beta rebound power, but not with beta frequency value. Compared to the value of peak frequency, the oscillation amplitude has been reported to be more closely associated with pharmacologically-induced GABAergic modulation^21, 27^, though some negative data have also been reported^28^. Taken together, the relationships between GABA and brain oscillations are under active investigation through different methodologies and our current results warrant further in-depth investigation.

Functionally, evidence suggests that the increase in beta power induced by median nerve stimulation is associated with decreased motor cortical excitability^29^. Using transcranial magnetic stimulation (TMS) at different conditioning-test (C-T) intervals, Chen and Colleagues found the amplitude of motor evoked potentials (MEP) to be significantly reduced at C-T intervals of 400, 600, and 1000 ms, with a maximum reduction at 600 ms^29^. The interval with maximum reduction corresponds to the peak latency of the beta rebound activity^29^. Thus, our data supports the notion that the electricity-induced beta power is associated with GABAergic inhibition and concomitant changes in cortical excitability.

Sensory gating paradigms have been widely used in the auditory modality to study early-stage perceptual processing related to higher-order, attentive inhibitory modulation^4, 30^. In addition, paired-pulse electrical stimulation of the median nerve has been successfully applied in the investigation of somatosensory SG^3, 7–9, 31, 32^. In a previous study of ours, an association between somatosensory SG and behavioral performance of inhibition control was found^3^, suggesting that the pre-attentive SG ability could be a useful imaging marker of cortical inhibitory function. This association together with the findings of the present study further suggests a close relationship between SI and MI in context of automatic inhibitory function albeit with MI changes being more closely related to changes in GABA+ levels.

In contrast to our expectation, the SI SG was not correlated with the cortical endogenous, resting GABA+ concentration. Interestingly, a non-significant result was also found in our previous work, in which the auditory cortex was studied^33^. Although the reason for the lack of such a link remains unclear, we speculate that an intermediate factor, e.g., a certain band of oscillations, cross-frequency coupling, etc., may obscure the association between SG and GABA+ levels. Another contributing factor could be that SI SG may rely on GABAergic mechanisms that are not visible to MRS GABA. It has been suggested that MRS is mainly sensitive to the GABAergic tone produced via extrasynaptic GABA_A_ receptors^34, 35^. Alternatively, a differential sensitivity of different neurotransmitters to the neurophysiological measures may also be a possibility. For example, a significant association was found between MRS-assessed glutamate levels and TMS measures of cortical excitability, while no relationship was found with TMS measures of inhibitory activity in MI^34^.

One might argue that the GABA+ value of Subject 3 served as a potential outlier driving the significant correlational results. However, for the following reasons we deduce that this high value of GABA+ should not be considered extreme: (1) it fell within 2.5 standard deviations from the mean, (2) no significant outliers were detected in our data using Grubb’s test, and (3) Subject 18, who was not included in the analysis due to missing data, exhibited similar GABA+ level. Despite this reasoning, we acknowledge that our data should be interpreted with caution and that the results would gain in significance by being reproduced with a larger sample size.

Finally, it should be noted that although we used the state-of-the art procedures to acquire and process the spectral data, the estimation and quantification of GABA+ at 3 Tesla is limited by the low signal-to-noise ratio and the relatively long acquisition time. Thus, GABA+ was obtained from a relatively large region including both SI and MI. In addition to the large and cuboidal MRS voxel, the curvature of the central sulcus also limited the anatomical specificity of the VOI, i.e. it was not possible to exclusively acquire data from either MI or SI. Although the GABA+ concentration was constrained to the sum of SI and MI signals, we still consider it reasonable to test its correlation with independent measures of motor and somatosensory function that were acquired by the electrophysiological recordings. Future studies could apply MRS with an increased field strength, such as 7 Tesla, to obtain increased regional specificity for the GABA measurement.

In summary, we have shown that SG in the SI region and beta rebound power in the MI region was significantly correlated, suggesting that SI and MI work in concert to serve automatic inhibitory function. Furthermore, the *in vivo* resting cortical GABA+ concentration is associated with the power of the motor cortical beta rebound but not the somatosensory SG, suggesting that GABA+ levels, as measured with MRS, are more closely related to the automatic inhibitory function of MI than SI.

## Methods

### Participants

Eighteen healthy right-handed volunteers (age 20–33 years, 5 females) participated in this study after giving informed consent. All the subjects had normal or corrected to normal vision and reported no neurological or psychiatric diseases. All the experimental procedures were approved by the Institutional Review Board of Taipei Veterans General Hospital (Taipei, Taiwan), and were performed in accordance with approved guidelines and regulations. An informed consent was obtained from each participant.

### MEG recordings and analyses

Two blocks of MEG recordings were acquired in a counterbalanced sequence. In one block, a paired-pulse paradigm was used to measure the SG ratio, and in the other block a single-pulse paradigm was used to estimate beta oscillatory activity.

Each subject sat comfortably in the MEG room with the head fully supported by the MEG helmet. In the paired-pulse paradigm, stimuli were delivered in pairs with an ISI of 500 ms and inter-pair interval of 6 s to the left median nerve. In the single-pulse paradigm, stimuli were delivered repeatedly with an ISI of 1.6–2.0 s. The stimulus intensity was set at 20% above the motor threshold to elicit a visible twitch of the thumb. During both blocks of MEG recordings, the subjects were instructed to focus on watching a silent movie and to ignore the stimuli.

Brain responses were continuously recorded by a 306-channel whole-head MEG (Vectorview, Elekta, Neuromag, Helsinki, Finland), with a sampling rate of 1000 Hz and an online bandpass of 0.1–200 Hz. Epochs with prominent electrooculogram (>300 μV) or MEG (>3000 fT/cm) artifacts were excluded from averaging. Only the MEG signals from the 204 gradiometers, which detect the largest signals above the activated areas^36^, were analyzed. At least 100 artifact-free trials in each paradigm were collected for further analyses.

All the MEG data were offline analyzed by using the Matlab-based open source toolbox Brainstorm^37^. For the data from paired-pulse electrical stimulation, the averaged epochs were further bandpass filtered (0.1–120 Hz) and baseline corrected using the 100 ms preceding S1 and S2. Cortically constrained source imaging was performed by using the depth-weighted minimum norm estimate (MNE)^38, 39^. A set of ~15,000 elementary current dipoles distributed over the cortical envelope was geometrically registered to the Montreal Neurological Institute (MNI) brain template Colin27. The MNE source maps were obtained from each subject and each stimulus condition (i.e., S1 and S2). In the present study, the P35m-component was selected to study the somatosensory SG because previous studies have shown a robust gating effect for this component^3, 7, 9^. For each participant’s largest cortical activation of P35m, a cluster of 30 vertices corresponding to 4–5 cm^2^, was manually identified in the SI. The magnitude of each elementary dipole was normalized to its fluctuations over the baseline, yielding a z score at each cortical location. The z values of the P35m cortical responses to S1 and S2 were then extracted to calculate the SG ratio (S2/S1).

For single-pulse stimulation, distributed MNE was also applied to reconstruct the source activation of electricity-induced beta oscillations. Since rolandic beta oscillations have been shown to originate from M1^13, 14^, a region of interest (ROI) was identified using a set of 30 vertices corresponding to 4–5 cm^2^ in MI, for each participant. In order to characterize the power spectrum, single trial source waveforms (100 ms before and 1000 ms after stimulation) extracted from the ROI were transformed using Morlet wavelet-based time-frequency analysis, with a central frequency of 1 Hz and a time resolution of 3 s. The time-frequency responses were computed and displayed between 1–50 Hz in 1 Hz steps. The mean strength of the most reactive beta oscillations (2 Hz for consecutive bins) was identified and calculated from the average of 200 ms centered around the peak latency (100 ms before and 100 ms after the peak). Also, z-normalized values were computed to obtain absolute magnitude changes with respect to baseline levels in each participant.

### MRS recordings and analyses

All experiments were performed with a 3 T MR system (TRIO, SIEMENS Medical Solutions, Erlangen, Germany) using a 32-channel phased-array head coil. A high-resolution 3D MPRAGE (Magnetization Prepared Rapid Acquisition Gradient Echo) anatomical scan (TR/TE/FA: 2530 ms/3.03 ms/7 degrees; FOV: 256 × 256 × 176; voxel size: 1 × 1 × 1 mm^3^) was initially acquired for localization of the spectroscopic volume of interest (VOI). The VOI was manually positioned at the right SM cortex, with the size of 25 × 25 × 25 mm. To ensure the quality of the spectra, a pre-scan was carried out using a point resolved spectroscopy (PRESS) sequence (TR/TE = 2000/68 ms, sample points = 2048, bandwidth = 2000 Hz, 16 averages). These spectra were analyzed and displayed online to evaluate the linewidth, water suppression and noise level. Once spectra in the pre-scan were considered to be of acceptable quality, GABA measurements were performed directly using the same adjustment parameters for shimming, resonance frequency and water suppression. A MEGA-PRESS sequence was used for GABA measurements^40^. A total of 300 spectra were acquired using the following parameters: TR/TE = 2000/68 ms, sample points = 2048, bandwidth = 2000 Hz, phase cycling steps = 4. The 300 spectra were acquired as 75 repeats of a 4-step phase cycle with the editing pulse set to edit-on and edit-off on alternate phase cycles. GABA-editing was achieved with a 15 ms Gaussian pulse applied at 1.9 ppm for edit-on spectra and at 7.5 ppm for edit-off spectra. In addition, a non-water suppression MRS scan was acquired to obtain an unsuppressed water signal for normalization. The total scan time for MRS acquisition was approximately 10 minutes.

For each subject, MRS data were saved separately and the first 4 spectra were discarded. This resulted in 148 edit-on spectra and 148 edit-off spectra. Data processing was performed in Matlab (The MathWorks, Natick, USA) using in-house made scripts^41^. Exponential line broaden filtering and phase correction were applied on each time-domain spectrum. To extract information of resonance frequency shifts during scans, the location of the creatine (Cr) peak at 3.01 ppm was identified and fitted with a Lorentzien model on each edit-off spectrum. Pairwise alignment was performed based on peak location of Cr for each edit-on and edit-off spectrum. Further phase correction was applied on the averaged edit-on spectrum and the averaged edit-off spectrum by aligning the shape of the residue water peak. The final spectrum was generated by subtraction of the averaged edit-off spectrum from the averaged edit-on spectrum. The signal at 3.0 ppm was linear baseline corrected and then quantified using fitting with a two-Gaussian model with 7 variables (2 height, width, frequency, splitting, baseline offset, baseline gradient) in the spectral range of 2.79–3.55 ppm^41, 42^. Since the quantified GABA signal also contains contributions from homocarnosine and macromolecules^19^, the GABA signal is designated as GABA+ throughout the manuscript. On the edit-off spectrum, the Cr signal at 3.03 ppm was fitted by a Lorentzian line shape function with 5 variables in the spectral range of 2.72–3.12 ppm. The water signal was also fitted by a Lorentzian line shape function but on the NWS spectra in the spectral range of 3.8–5.6 ppm. The GABA+ signal was then normalized to the water signal using the water scaling method. To perform a partial volume correction, the MPRAGE image was segmented into gray matter (GM), white matter (WM) and cerebral spinal fluid (CSF) using SPM8 (www.fil.ion.ucl.ac.uk/spm). Separate images of the three tissue types were generated and percentages of GM, WM and CSF were determined for the VOI. GABA+ signals were then corrected for partial volume and relaxation effects based on tissue percentages and previously reported T1 and T2 values^41–43^.

### Statistical analyses

All the data were presented as mean ± standard error of the mean (SEM) (IBM SPSS Statistics, version 19). Each parameter was evaluated with Kolmogorov-Smirnov one-sample test, and the results showed the data to be normally distributed (all p values > 0.05). We, therefore, applied parametric analyses for hypothesis testing. The Pearson’s correlation coefficient tests were primarily used to evaluate the following linear relationships: (1) SI SG ratio and MI beta rebound power, (2) SM cortical GABA+ levels and MI beta rebound power, (3) SM cortical GABA+ concentration and MI beta rebound peak frequency, and (4) SM cortical GABA+ concentration and SI SG ratio. Additional analyses of Spearman’s rank correlation coefficient tests were also performed. Adjusted p-values of 0.05, corrected for multiple comparisons by the Benjamini and Hochberg approach^44^, were set as the significance level and reported in the present study.

## Electronic supplementary material

Supplementary Figure
