# Supplementary material for: Automatic inhibitory function in the human somatosensory and motor cortices: An MEG-MRS study
Source: Sci Rep. 2017 Jun 26;7:4234. doi: 10.1038/s41598-017-04564-1 (PMC5484662; doi:10.1038/s41598-017-04564-1)
Supplement: Supplementary file 1 — Supplementary Figure [file 41598_2017_4564_MOESM1_ESM.doc]

Scientific Reports - Electronic Supplementary Information

**Automatic inhibitory function in the human somatosensory and motor cortices: An MEG-MRS study**

Chia-Hsiung Cheng^1,2,3,4^*, Shang-Yueh Tsai^5,6^, Chia-Yih Liu^4,7^, David M. Niddam^8,9^*

1. Department of Occupational Therapy and Graduate Institute of Behavioral Sciences, Chang Gung University, Taoyuan, Taiwan
2. Laboratory of Brain Imaging and Neural Dynamics (BIND Lab), Chang Gung University, Taoyuan, Taiwan
3. Healthy Aging Research Center, Chang Gung University, Taoyuan, Taiwan
4. Department of Psychiatry, Chang Gung Memorial Hospital, Linkou, Taiwan
5. Graduate Institute of Applied Physics, National Chengchi University, Taipei, Taiwan
6. Mind, Brain and Learning Center, National Chengchi University, Taipei, Taiwan
7. School of Medicine, Chang Gung University, Taoyuan, Taiwan
8. Institute of Brain Science, National Yang-Ming University, Taipei, Taiwan
9. Brain Research Center, National Yang-Ming University, Taipei, Taiwan

Corresponding author:

Chia-Hsiung Cheng, Ph.D.

Department of Occupational Therapy and Graduate Institute of Behavioral Sciences, Chang Gung University, Taoyuan, Taiwan

Address: No. 259, Wenhua 1^st^ Rd., Taoyuan City 333, Taiwan

Email: [ch.cheng@mail.cgu.edu.tw](mailto:ch.cheng@mail.cgu.edu.tw), chiahsiung.cheng@gmail.com

Tel: +886-3-2118800 #3854

Fax: +886-3-2118700

and

David M. Niddam, Ph.D.

Brain Research Center, National Yang-Ming University, Taipei, Taiwan

Address: No.155, Sec.2, Linong St., Taipei, 112 Taiwan

Email: niddam@ym.edu.tw


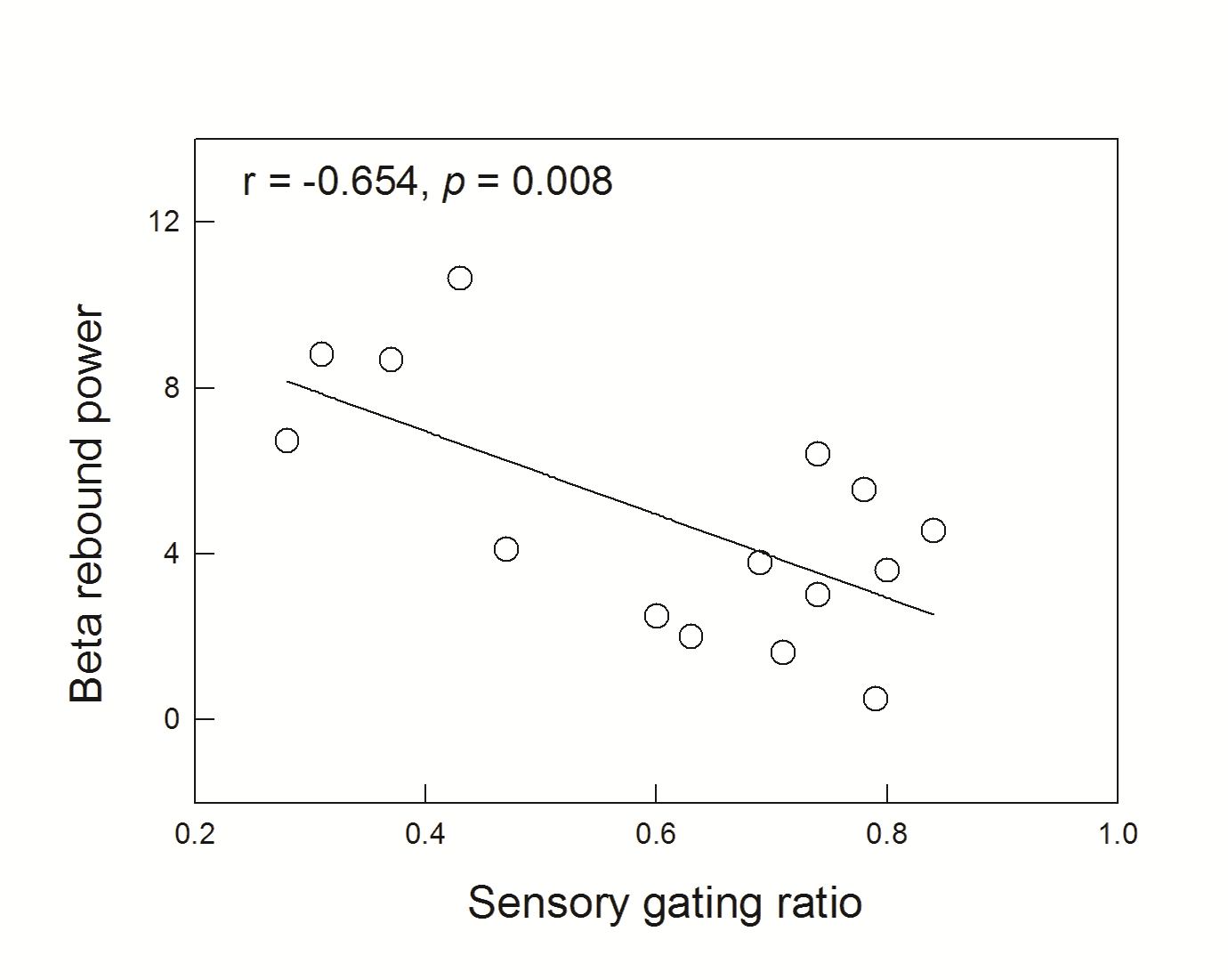


**Supplementary Figure.** Since the beta rebound begins from around 400 ms after the S1, it might be argued that this oscillatory response possibly elicits more fluctuations in the 100 ms baseline before the S2 in the current paired-pulse paradigm with an ISI of 500 ms. Such an effect likely leads to a reduced z score even without actual differences in the S2 response. Thus, we also reported the sensory gating (SG) ratios that were obtained from the absolute values of S1 and S2 without z normalization. As shown in the scatter plot, the SI SG ratio, calculated from the absolute value of S1 and S2, was still significantly correlated with the MI beta rebound power.
